# The patient needing prolonged mechanical ventilation: a narrative review

**DOI:** 10.1186/s40248-018-0118-7

**Published:** 2018-02-26

**Authors:** Nicolino Ambrosino, Michele Vitacca

**Affiliations:** 1 Istituti Clinici Scientifici Maugeri, IRCCS, Istituto Scientifico di Montescano, 27040 Montescano, PV Italy; 2 Istituti Clinici Scientifici Maugeri, IRCCS, Respiratory Unit, Istituto Scientifico di Lumezzane, Lumezzane, BS Italy

**Keywords:** Artificial nutrition, Critical illness, Intensive care unit, Mobilisation, Noninvasive mechanical ventilation, Respiratory failure, Tracheostomy, Weaning mechanical ventilation, Weaning units

## Abstract

**Background:**

Progress in management has improved hospital mortality of patients admitted to the intensive care units, but also the prevalence of those patients needing weaning from prolonged mechanical ventilation, and of ventilator assisted individuals. The result is a number of difficult clinical and organizational problems for patients, caregivers and health services, as well as high human and financial resources consumption, despite poor long-term outcomes. An effort should be made to improve the management of these patients. This narrative review summarizes the main concepts in this field.

**Main body:**

There is great variability in terminology and definitions of prolonged mechanical ventilation.

There have been several recent developments in the field of prolonged weaning: ventilatory strategies, use of protocols, early mobilisation and physiotherapy, specialised weaning units.

There are few published data on discharge home rates, need of home mechanical ventilation, or long-term survival of these patients.

Whether artificial nutritional support improves the outcome for these chronic critically ill patients, is unclear and controversial how these data are reported on the optimal time of initiation of parenteral vs enteral nutrition.

There is no consensus on time of tracheostomy or decannulation. Despite several individualized, non-comparative and non-validated decannulation protocols exist, universally accepted protocols are lacking as well as randomised controlled trials on this critical issue. End of life decisions should result from appropriate communication among professionals, patients and surrogates and national legislations should give clear indications.

**Conclusion:**

Present medical training of clinicians and locations like traditional intensive care units do not appear enough to face the dramatic problems posed by these patients. The solutions cannot be reserved to professionals but must involve also families and all other stakeholders. Large multicentric, multinational studies on several aspects of management are needed.

## Background

The increasing worldwide life expectancy results in high prevalence of patients suffering from chronic diseases and related “chronic critical illness” [1, 2]. Up to 20 million people annually require Intensive Care Units (ICUs) admission and mechanical ventilation (MV). The progresses in management of these patients has improved their short-term survival at the price of a growing population of patients with partial or complete dependence on MV. The prevalence of these ventilator assisted individuals (VAIs) ranges from 6.6 to 23 per 100,000 [3–5] resulting in difficult clinical and organizational problems for patients, caregivers and health services, as well as in high human and financial resources consumption, despite poor long-term outcomes [6, 7]. In order to minimize the VAI prevalence an effort should be made to improve the management of patients needing weaning from prolonged MV (PMV) [2]. This narrative review of available literature summarizes the main concepts in this field with the aim to update the knowledge of professionals caring respiratory patients on this emerging problem.

## Main text

This article is a narrative review of randomized controlled trials (RCTs), observational studies, systematic reviews, and meta-analyses published between 1990 and 2017 in English, in PubMed, and Scopus databases using the keywords: Cronic Critical Illness, Noninvasive mechanical ventilation, Mechanical ventilation, Physiotherapy and ICU, Tracheostomy, Ventilator Assisted Individuals, Weaning mechanical ventilation, Weaning Unit. In the following pages the terms “Prolonged Weaning” and “PMV” will be used with the same meaning.

### Definitions and epidemiology

#### Chronic critical illness

A consensus-derived definition of patients suffering from chronic critical illness, included patients with one of six eligible clinical conditions: PMV, tracheostomy, stroke, traumatic brain injury, sepsis or severe wounds, and at least 8 days of ICU length of stay [8]. The study [8] reported that 7.6% of patients admitted to an ICU met these inclusion criteria, with a 30.9% hospital mortality. Many survivors may suffer from persisting physical disabilities, and reduced quality of life, even years after discharge from the ICU [9, 10]. Several problems may contribute to these limitations.

Diaphragm weakness is highly prevalent in critically ill patients. It may exist prior to ICU admission and may induce the need for MV but it also frequently develops during the ICU stay. Several risk factors for diaphragm weakness have been identified, among them sepsis and length and modalities of MV play central roles. Critical illness-associated diaphragm weakness is consistently associated with poor outcomes including increased ICU mortality, difficult weaning and PMV [11, 12].

A long hospital stay and lack of response to or an inadequate level of appropriate therapy can lead to muscle wasting and weakness, deconditioning, recurrent symptoms and mood alterations [13]. There are also substantial derangements of the hypothalamic-anterior pituitary-peripheral hormonal axes [14]. Subjects under PMV may show a lower hypercapnic ventilatory response than successfully weaned subjects [15]. A focus must be addressed also to sleep disturbances in the ICU due to the possible link between deprived sleep and development of delirium, prolonged stay in the ICU, and increased mortality [16]. Factors associated with prolonged weaning are summarized in Table 1.

**Table 1:** Factors associated with prolonged mechanical ventilation

• **Systemic**	
	**○** Chronic diseases, comorbidities,
	**○** Nutrition and metabolic problems
	**○** Severity of illness
	**○** Sepsis
• **Cardio-vascular function**	
• **Critical Illness Neuromyopathy**	
• **Respiratory**	
	**○** Unresolved respiratory causes of respiratory failure
	**○** Diaphragm weakness or dysfunction
	**○** Imbalance between work of breathing and respiratory muscle reserve
	**○** Tracheo-bronchial obstruction
	**○** Ineffective cough and secretion retention
• **Complications of management.**	
	**○** Ventilator associated pneumonia, infection
	**○** Length and modalities of mechanical ventilation
	**○** Tracheostomy
	**○** Sedation
	**○** Lack of early mobilizatio
• **Cognitive**	
	**○** Sleep deprivation
	**○** Anxiety/Depression
• **Management setting**	
	**○** Protocols
	**○** Staffing, (number and professionals)
	**○** Personnel training

#### Prolonged mechanical ventilation/prolonged weaning

There is great variability in terminology and definitions [17]:

- *National Association for Medical Direction of Respiratory Care (NAMDRC)* [18]: “the need for more than 21 consecutive days of MV for more than 6 h/day”.
- *European Respiratory Society (ERS) Task Force* [19]: “the need of more than 7 days of weaning after the first spontaneous breathing trial (SBT)”. According to this definition patients may represent up to 14% of those admitted to ICU for MV, accounting for 37% of all ICU costs with a hospital mortality up to 38% [20], significantly higher compared with simple and difficult weaning [20–22].
- *Weaning according to New Definition (WIND) study* [23]: “successful extubation after more than three SBTs or taking more than seven days”. According to this definition, PMV accounts for 10% of patients receiving MV with a 29.8% mortality [23].

A population-based cohort study [6] in an ICU in Canada, reported that 5% of patients underwent PMV, with 42% hospital mortality vs 28% of non PMV patients. Among hospital survivors, estimated 1- and 5- year mortality for PMV patients was 17% and 42% respectively. A recent systematic analysis [24] of literature on long-term survival of PMV patients reported a 59–62% mortality at 1 year. Pooled mortality at hospital discharge was 29%. However, only 19% were discharged home and only 50% were successfully liberated from MV [24].

### Strategies for successful weaning

There have been recent progresses in weaning from MV, among which:

- ***Ventilatory strategies***;
- ***Weaning protocols***;
- ***Early mobilisation and Physiotherapy***,
- ***Specialised weaning units***.

#### Ventilatory strategies

The most popular ventilatory strategies used to shorten and achieve a more succesful weaning from MV in the ICU are [25]:

- Progressive reduction in the level of assistance of Pressure Support Ventilation (PSV);
- Progressive longer periods of SBT through the tube;
- Syncronized Intermittent Mandatory Ventilation (SIMV: the patient can breath spontaneously between ventilator-delivered breaths).
- Neurally Adjusted Ventilatory Assist (NAVA).
- Noninvasive mechanical ventilation (NIV).
- High-flow oxygen (HFO).

In the Nineties multicentric comparative studies in the ICUs gave conflicting results, reporting advantages with either PSV [26], or SBT [27], or equivalent results [28]. In both studies [26, 27] SIMV was the least effective modality. A meta-analysis evaluated the studies comparing PSV and SBT [29]: the effects on weaning success, ICU mortality and reintubation rates, ICU and long-term weaning unit (LWU) length of stay, and pneumonia were imprecise.

Neurally Adjusted Ventilatory Assist is a mode recently introduced in the clinical use in which the ventilator applies an inspiratory positive pressure in proportion to the electrical activity of the diaphragm, the best available indicator of the neural drive to breathe [30]. This modality has been used during weaning from MV in ICU patients and, compared to PSV, resulted in reduced patient-ventilator asynchronies, and in a breathing pattern more similar to spontaneous ventilation [31].

Nava et al. [32] were the first to use NIV to shorten time of weaning from invasive MV in patients with acute respiratory failure (ARF) due to acute exacerbations of chronic obstructive pulmonary disease (AECOPD), while avoiding the complications of invasive MV. Noninvasive mechanical ventilation during weaning was as effective as invasive MV in improving breathing pattern, reducing the work of breathing (WOB) with adequate gas exchange [33]. The recent ERS/American Thoracic Society (ATS) guidelines [34] suggest that NIV*:*

- Should be used to facilitate weaning from MV in patients with hypercapnic ARF only in centres with adequate experience using NIV in this setting [35].
- Should not be used in the treatment of patients with established post-extubation ARF.
- Should be used to prevent post-extubation ARF in high-risk patients but not used to prevent post-extubation ARF in non-high-risk patients.

More recently, the use of HFO compared with conventional oxygen therapy reduced the risk of re-intubation within 72 h in extubated patients at low risk for re-intubation [36]. Among high-risk extubated adults conditioned HFO was not inferior to NIV in preventing re-intubation and post-extubation ARF [37].

### Which ventilatory strategy does better work in PMV patients?

#### PSV vs SBT

A prospective multicenter RCT [38] in three LWUs evaluated which protocol, progressive reduction in the level of assistance of PSV or progressive longer periods of SBT through the tube, was more effective in weaning COPD patients requiring MV for more than 15 days. No significant difference was found in weaning success and hospital mortality rate, duration of ventilatory assistance, LWU and hospital length of stay. Jubran et al. [39] found that the use of the SBT protocol with a tracheostomy collar resulted in shorter median weaning time, without any effect on 6- and 12- month survival. The shorter weaning time with the SBT protocol with tracheostomy was attributed to its effect on clinical decision making. It was supposed that, with this modality the WOB is sustained only by the patient, and as such, observing a patient breathing through a tracheostomy would provide the clinician with a clear view of the patient’s respiratory capacities. In contrast, authors argued that the ability to judge weanability during the PSV protocol is reduced because the patient is receiving ventilator assistance [40].

#### Neurally adjusted Ventilatory assist

A study in PMV patients [41], confirmed that NAVA eliminates the risk of overassistance. However, it also indicated that the advantages of NAVA over PSV were smaller when PSV was carefully set avoiding excessive support. However whether NAVA may help to speed up, the prolonged weaning of these PMV patients requires further studies [42].

#### Noninvasive ventilation

A prospective study included chronically critically ill patients admitted to Spanish respiratory care units [43]. The weaning method consisted of progressive periods of SBT. Patients were transferred to NIV when it proved impossible to increase the duration of SBT beyond 18 h. Eighty-six % of patients were successfully weaned, out of whom 21% needed NIV during the weaning process. Some authors suggest that many VAIs, especially patients suffering from neuromuscular diseases, can be decannulated even in outpatient clinics to continuous NIV with the aid of mechanical insufflation-exsufflation [44]. In particular it is suggested that patients with myopathic or lower motor neuron disorders might be managed by NIV, indefinitely, despite having little or no measurable vital capacity, with the use of respiratory muscle aids [45].

#### Weaning protocols

Trials have demonstrated that application of protocols or guidelines for the weaning process may lead to a decrease in weaning time independent of the mode used, even better than automatic systems [46, 47]. The ATS/CHEST guidelines suggest to use ventilator liberation protocols to manage adults mechanically ventilated for > 24 h [48].

In patients needing PMV a well defined protocol, independent of the modality used, was associated with a better outcome than uncontrolled clinical practice previously performed in the LWUs [38]. A multifaceted strategy consisting of continuing education and regular feedback can increase physician adherence to a weaning protocol for MV [49].

#### Early mobilization and physiotherapy

Overall, approximately 25% of PMV patients in the ICU develop generalized and persistent muscle weakness: approximately one million patients develop the ICU-acquired weakness syndrome (critical illness neuromyopathy) annually [50]. Muscle deconditioning occurs very early with bed rest, involving more calf and other antigravity muscles, than other muscles, such as those involved in the grip strength. Muscle atrophy is associated with decline in muscle mass, strength and aerobic efficiency, and has been reported that the predominant muscle composition changes from type IIa, with higher aerobic capacity, to type IIb fibres [51]. ICU-acquired weakness worsens acute morbidity, increases healthcare related costs and 1-year mortality. Persistence and severity of weakness at ICU-discharge further increases 1-year mortality [52].

**Fig. 1:**
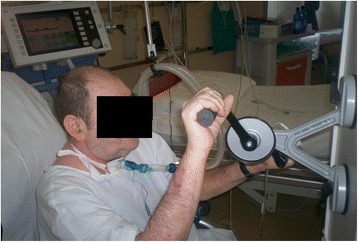
A tracheostomized patient performing upper arm exercise under MV

Evidence of benefits from early mobilization and physiotherapy has progressed during the past 15 years with RCTs, systematic reviews [53, 54], and recommendations [55] including mobilization [56] and muscle electrical stimulation protocols [57]. For adults mechanically ventilated for > 24 h, the ATS/CHEST guidelines suggest to use protocols of early mobilization, without any superiority of a protocol over another [48, 58]. Figure 1 shows a tracheostomized patient performing upper arm exercise training under MV.

Ineffective cough and secretion retention can play a significant role in weaning failure. Evaluation of cough strength by means of assessment of cough peak expiratory flow rate can predict extubation failure and may reduce ICU length of stay, expenditures, morbidity and mortality [59]. Cough augmentation techniques, such as lung volume recruitment or manually- and mechanically-assisted cough, are used to facilitate extubation and prevent post-extubation respiratory failure. However the quality of studies is very low, leading only to conclusion that these techniques when used in patients under MV result in few adverse events [60]. However, despite evidence and recommendations, there is still limited awareness of the clinical benefits of early mobilization and physiotherapy techniques and high level of disagreement on the sustainable maximal level of activity in these critically ill patients. Several factors (e.g. multidisciplinary rounds, setting daily goals for patients, 7/7 day availability of dedicated physiotherapists, and nurse/patient staffing ratio) are significantly associated with the practice of early mobilization in ICU and international structures and practices are heterogeneous [61]. Physiotherapy techniques commonly used for early mobilization and airway clearance are shown in Table 2.

**Table 2:** Physiotherapy activities and techniques for patients with prolonged mechanical ventilation

• **Muscle weakness**	
	**○** Passive and active-assisted mobilisation
	**○** Continuous rotational therapy
	**○** Postures
	**○** Active limb exercise
	**○** Peripheral muscle training
	**○** Neuromuscular electrical stimulation
	**○** Respiratory muscle training
• **Cough augmentation techniques**	
	**○** Manual hyperinflation
	**○** Percussion and vibrations
	**○** Mechanical In-Exsufflation
	**○** Percussive ventilation

#### Specialised weaning unit

To take care of difficult-to-wean patients, recently the problem of appropriate ICUs utilisation has been faced by proposing different locations and modalities of assistance [62]:

- Respiratory intermediate Intensive Care Units (RIICUs) within acute care hospitals [63] are less costly than ICUs, but usually offer adequate levels of assistance and may also provide multidisciplinary rehabilitation. Some of these RIICUs may work also as “step down” units for PMV patients serving also as a bridge to home-care programs or long-term care facilities [63].
- Alternatively patients needing PMV may be transferred from acute care hospitals to specialised regional LWUs, often located within rehabilitation hospitals. These dedicated LWUs relieve pressure on ICU beds at lower costs, with specialized teams (e.g. nurses, respiratory therapists, nutritionists, psychologists, speech and occupational therapists). Variable mortality and weaning success rates have been reported [64–66].
- More recently modalities of tele-monitoring have been proposed as a means to manage difficult-to-wean patients and VAIs [67].

### Long-term outcomes

There are few published data on discharge home rates, use of NIV, or long-term survival in specialized LWUs. An early study [68] evaluated survival and weaning success rate in COPD patients needing PMV (more than 21 days) in an Italian LWU. Fifty-five percent of patients were successfully weaned with a 68% overall 2- year survival rate in weaned as compared to 40% in unweaned patients. A database review [69] of patients admitted to a UK LWU reported that 91% of patients transferred for weaning from PMV survived to hospital discharge. Seventy-two% of patients were weaned, weaning success rate being highest for patients with COPD and chest wall disorders. Median survival from LWU discharge was 25 months [69]. In another report [63], 49 tracheostomized difficult-to wean patients were transferred from ICUs to a University-Hospital RIICU after a mean ICU length of stay of 33 days. The weaning success rate in the RIICU was 67% with a mean length of stay of 17 days. Ten per cent of patients died, 20% failed weaning and were transferred to a dedicated LWU where 60% were weaned. The overall weaning success rate of this model (RIICU+LWU) was 80%, with 16% and 5% hospital and 3- month mortality respectively. The model resulted in cost saving per patient compared to ICU [63]. A retrospective analysis [66] of the characteristics and outcomes for patients consecutively admitted to a LWU after cardiac surgery between 2007 and 2012 reported that compared with patients with single cardiac intervention, patients undergone combined cardiac interventions showed a significantly lower successful weaning rate (44% vs 79%) and a higher hospital mortality (31% vs 5%). The overall 6- month survival for single intervention patients was 74% compared with 37% for the other patients [66]. Another analysis [65] in five Italian LWUs comparing three periods of 5 consecutive years (from 1991 to 2005) on more than 3,000 patients reported that the overall weaning success rate decreased (from 87% to 66%), and the mortality rate increased over time (from 9 to 15%) [65]. It is interesting to evaluate what happens to these patients needing PMV where there is no LWU. A prospective prevalence study [70] in 55 chinese ICUs, with 28 days follow up, reported that 36.1% of patients had received MV for more than 21 days, 23% of them being weaned. Despite the public health burden required by patients requiring PMV, a systematic review [71] reports that only 14 articles in the biomedical literature have tested patient-level factors associated with long-term mortality. Six factors demonstrated strong evidence for association with mortality: age, vasopressor requirement, thrombocytopenia, preexisting kidney disease, failed ventilator liberation, and acute kidney injury, hemodialysis requirement [71]. Other predictive factors have been proposed: clinical variables available on day 14 of MV, the ProVent 14 model, could identify patients receiving PMV with a high risk of mortality within 1 year [72].

### Nutrition

Malnutrition in critically ill patients is associated with poor outcomes, including impaired wound healing, higher rates of nosocomial infections, and all-cause mortality [73]. Nutritional status of these patients is influenced by both chronic and acute starvation, but also by the severity of the underlying pathophysiological processes leading to ICU admission. There is a marked catabolic response leading to rapid loss of lean body mass, varying from 5% in single-organ failure to 25% in multi organ dysfunction syndrome during the first days of admission [51]. In tracheostomized difficult-to wean patients natural meals may induce an increase in WOB [74].

Whether artificial nutritional support improves the outcome for critically ill patients is unclear [75]. The administration route, the time of starting artificial nutrition, the number of calories, and the type of nutrients seem to be important [76]. Controversial data are reported on the optimal time of initiation of parenteral vs enteral nutrition. In a study, late initiation of parenteral nutrition was associated with faster recovery and fewer complications, as compared with early initiation [77]. A later study reported that in critically ill adults with relative contraindications to early enteral nutrition, early parenteral nutrition resulted in significantly fewer days of invasive MV but not significantly shorter ICU or hospital length of stay or in 60-day mortality as compared with standard care [78]. Percutaneous endoscopic gastrostomy (PEG) is the preferred measure to deliver enteral nutrition in the long-term setting including VAIs. Studies have shown the advantages of PEG over nasogastric tube in patients with dysphagia and neurological diseases [79].

### Tracheostomy and decannulation

Use of tracheostomy seems to increase in patients needing PMV [80] and is not associated with major discomfort even when using speaking valves [81]. However, early tracheostomy provided no benefit in terms of time of MV and hospital length of stay, mortality or infectious complications rates, and long-term quality of life in patients requiring PMV after cardiac surgery, although, the well-tolerated procedure was associated with less need of sedation, better comfort, and earlier resumption of autonomy [82].

Decannulation is the final step of liberation from PMV. Despite several individualized, non-comparative and non-validated decannulation protocols exist, universally accepted protocols are lacking as well as RCTs on this critical issue. Presence of an intact sensorium, coordinated swallowing and protective coughing are often the minimum requirements for a successful decannulation [83]. Also a survey in Italian RIICUs showed that, despite few complications of tracheostomies, there was no agreement on indications and systems for closure and that a substantial proportion of patients maintained the tracheostomy despite not requiring MV any more [84]. There is the need of accepted protocols for time and modalities of decannulation, also in the view that lack of decannulation of conscious tracheostomized patients before ICU discharge to the general ward was associated with higher mortality [85].

### Patients and families perceptions

In a prospective survey [86] in patients needing PMV, 82% of hospital survivors had transitions to postdischarge care locations, including 67% needing at least one readmission. Patients spent 74% of all their days alive in a hospital or a postacute care facility or under home care. At 1 year, only 9% of patients were alive without any functional dependency, 26% were alive with moderate dependency, and 65% were either alive with complete functional dependency or were dead. Patients with poor outcomes were older, had more comorbidities, and were more frequently discharged to a postacute care facility. The mean cost per patient was US$ 306,135. These severe outcomes are significantly worse than expectations of patients’ surrogates and physicians [87]. When PMV leads to a VAI the impact on patients, families and care-givers is relevant [88]. The family’s perception of care in patients under home MV during the last 3 months of life was reported in an Italian survey [89]. The majority of patients complained of dyspnoea and were aware of the severity and prognosis of the disease. Family burden was high especially in relation to money need [89].

### End of life management

These patients may require ethical end of life decisions such as withholding or withdrawing MV, appropriate symptoms management and adequate location outside the acute care hospital. Duration of weaning attempts and ICU length of stay should be defined on the basis of potential expected benefits: otherwise the lack of these elements may lead to a condition of treatment “oriented towards futility”. Appropriate palliative care for these patients has been associated with improved quality of life and reduction in intensive life-sustaining treatments [90] with improvement in caregivers’ psychological symptoms [91]. Unfortunately, the delivery of palliative care (other than pain assessment and management) is infrequent [92]. In European RIICUs and high dependency units, an end of life decision was taken only in 21% of patients [93]. Treatment withholding, do-not-intubate/do-not resuscitate orders and NIV as ventilatory care ceiling were the most common procedures. Competent patients and nurses were often involved [93]. Studies have evaluated interventions to improve the quality of palliative care in the ICU such as routine ethics, palliative care consultation, and optimal communication between ICU clinicians and families [94]. The management of patients admitted with treatment limitations varies dramatically among different ICUs. Among survivors, escalations are more common than de-escalations in aggressiveness of care [95]. Unfortunately, family-reported quality of end of life care for patients with cancer and those with dementia, was significantly better than for cardiopulmonary patients, mainly due to higher rates of palliative care consultation, more frequent do-not-resuscitate orders and fewer deaths in the ICUs [96]. Despite prognostication is a frequent question posed by family to decide whether to forego life support, there is evidence that physicians do not discuss the patient’s prognosis for survival in more than one third of conferences [97] or do not initiate discussions about palliative and end of life care with their patients [98]. It has been suggested that the “shared decision” approach may be the best one for end of life decision with respect for the autonomy of patients. Regional attitudes in Europe are different as physician’s religion sometimes influences end of life practice [99]. Life-supporting therapies were withheld or withdrawn in 11% of patients in French ICUs [100]. Futility and poor expected quality of life were the most frequently cited reasons. Decisions were mostly taken by all the ICU medical staff, with or without the nursing staff. The patient’s family was involved in the decision-making process only in 44% of cases [100]. Less than 15% of ICU patients retain decision making capacity, most patients have not completed advance written directives, the majority of patients have not discussed with relatives the preferences related to end of life care [101]. As a consequence, surrogate decision making occurs for nearly half of hospitalized older adults and includes both complete decision making by the surrogate and joint decision making by the patient and surrogate [102]. Terminal weaning is the gradual decrease in the level of ventilatory assistance. Compared to this modality, immediate extubation was not associated with differences in psychological effects on relatives if they perceived the modality as a standard practice in the ICU [103].

### Future research

There is a lack of wide scientific investigation in the field of prolonged mechanical ventilation. Future research should involve a shared definition of this condition for appropriate recruitment of patients. Large, multicentric, multinational RCT should be promoted and supported. We need studies on pathobiology and pathophysiology of these patients as distinct from acute critical illness patients. We also need well designed clinical RCT of different protocols of management on issues such as setting of MV, patient-ventilator interfaces, time of decannulation, nutritional support, sedation, drug therapy of symptoms and delirium, physical, psychological and cognitive long-term effects of prolonged mechanical ventilation. Costs/benefits of different facilities for care of these patients should be also evaluated in the frame of different health care systems (Table 3).

**Table 3:** Areas of future research

• Accepted definition of prolonged mechanical ventilation	
• Pathobiology and pathophysiology	
• Best setting of mechanical ventilation	
• Indications, time and modalities of decannulation	
• Nutritional support	
• Symptom management protocols	
• Best sedation protocols	
• Physical, psychological and cognitive long-term effects	
• Early mobilization protocols	
• Costs/benfits of different facilities	

## Conclusions

The clear side of progresses in management of ICU patients is improvement of hospital survival. The dark side is the increase in the number of difficult-to wean patients and related VAIs. Present medical training of clinicians and locations like ICU do not appear enough to face the dramatic problems (clinical, economical, ethical, legal) posed by these patients. Therefore the solutions cannot be reseved to professionals but must involve also families and all other stakeholders. We hope that this narrative review may contribute to offer patients, their families and associations, their caregivers and all interested stakeholders the occasion to sensitize governments and health services for the best management of these patients.
